# Systematic review and meta-analysis of the use of micrografting technology in humans

**DOI:** 10.1177/03000605251337859

**Published:** 2025-05-24

**Authors:** Rawan Almujaydil, Yumeng Yan, Sandra Kuswandani, Faisal Alotaibi, Jeanie Suvan, Linh Nguyen, Francesco D’Aiuto

**Affiliations:** 1 Periodontology Unit, UCL Eastman Dental Institute, UCL, UK; 2 Periodontology Unit, College of Dentistry, Qassim University, Saudi Arabia; 3 Department of Oral and Maxillofacial Surgery and Diagnostic Sciences, College of Dentistry, Prince Sattam bin Abdulaziz University, Saudi Arabia; 4 Oral Sciences, University of Glasgow Dental School, School of Medicine, Dentistry and Nursing, College of Medical, Veterinary and Life Sciences, University of Glasgow, UK; 5 Biomaterials and Tissue Engineering, UCL Eastman Dental Institute, UK

**Keywords:** Micrografting, regeneration, Rigenera, technology, Meek

## Abstract

**Aim:**

To critically assess the evidence on micrografting technology to evaluate its effectiveness when used alone or as an adjunct to regenerative treatment in various medical and dental applications.

**Methods:**

Seven electronic databases, including Cochrane Central Register of Controlled Trials (CENTRAL), Medline Ovid, Embase Ovid, Cumulative Index to Nursing and Allied Health Literature EBSCOhost, Web of Science Core Collection, System for Information on Grey Literature in Europe, and Bielefeld Academic Search Engine, were searched until 15 July 2024. Risk of bias assessment and qualitative and quantitative (random-effect models) analyses were conducted.

**Results:**

A total of 55 studies were identified. Most studies (n = 24) reported on burns, followed by 10 studies on ulcers/wounds, 7 on androgenetic alopecia, 3 on vitiligo, 3 on cartilage and bone defects, and 1 on coronary artery bypass graft surgery. Dental applications included sinus lift (three studies), socket preservation (two studies), and intrabody defects (two studies). A meta-analysis of four studies on the management of burns confirmed that micrografting led to reduced healing periods compared with other grafting techniques (weighted mean difference: −0.98, 95% confidence interval: −1.84 to −0.12, p = 0.03), with a high level of heterogeneity (83.57%) and risk of bias.

**Conclusion:**

Micrografting technology may lead to shorter healing time and improved patient morbidity.

## Introduction

Contemporary tissue regeneration approaches relying on surgical interventions continue to face substantial challenges, such as the scarcity of donor tissues available for transplantation and the associated morbidity resulting from invasive procedures.^1,2^ Indeed, advances in regenerative medicine have provided new options to recreate damaged/lost tissues. For instance, tissue-engineered constructs can be used as bone grafts for complex skeletal defects. Additionally, combining cells, scaffolds, and bioactive factors to create functional bone is a promising strategy, particularly in the development of hybrid materials with biomimetic and mechanical properties.^3^

Ten years ago, Human Brain Wave (HBW) developed the Rigenera technology and protocol for micrografting using the Meek technique concept. This medical device, called Rigeneracons, can extract tissue fragments from a person’s own tissue sample to improve regenerative outcomes in various clinical applications. The Rigenera micrografting technology has proven its effectiveness in various clinical scenarios. Moreover, it has been evaluated as a type of stem cell therapy, although the findings are controversial. Micrografting, an emerging technology for tissue regeneration, entails the application of small pieces of autologous healthy tissues to affected areas,^4^ aiming to enhance tissue regeneration, thereby reducing the morbidity of the harvesting process. Micrografting technique involves cutting a skin graft into smaller “micrografts” to increase its surface area, thereby covering a larger wound area than that of the original donor site. Micrografting was initially used to treat burns due to a shortage of available donor sites for skin grafting.^5,6^ In 1993, Kreis et al. improved the Meek technique by using a dedicated harvesting tool known as a dermatome with compressed air. This modification allowed the creation of larger postage stamp autografts. When combined with cultured grafts or allografts, the technique significantly improved clinical outcomes for severe skin burns, covering up to 75% of the wound.^7^ Stem cells are characterized by their capacity for self-renewal and the ability to differentiate into any type of cells, whereas micrografts are derived from a small piece of autologous tissue and have limited potential for differentiation compared with stem cells.^6^

Varying evidence exists regarding the techniques available; however, there has been no comprehensive assessment of the effectiveness of micrografting technology in several clinical applications. Therefore, this systematic review aimed to critically assess the available evidence regarding the effectiveness of micrografting technology in regenerative treatments.

## Material and methods

### Protocol and registration

**Figure 1.**
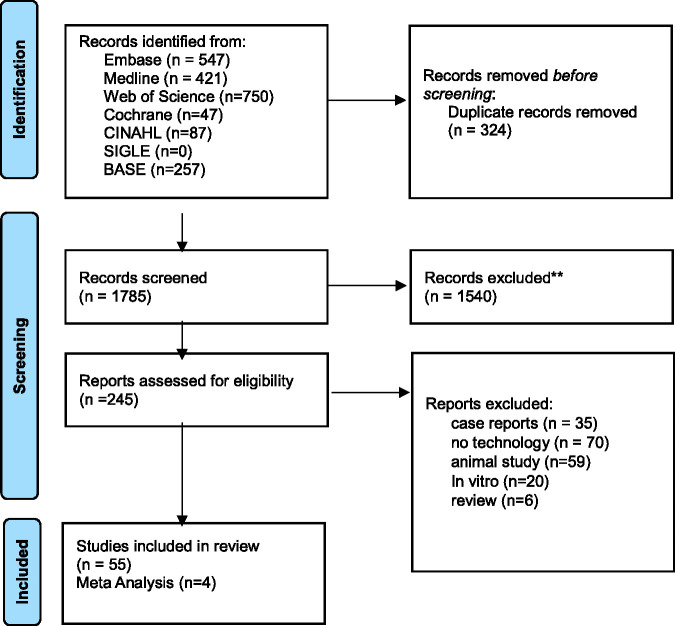
Preferred Reporting Items for Systematic reviews and Meta-Analyses (PRISMA) flow diagram of the studies.

This review followed the Preferred Reporting Items for Systematic Reviews and Meta-Analyses (PRISMA) checklist (Figure 1),^8^ and the study protocol was registered in the PROSPERO database (ID number: CRD42022332302).

### Patient/population, intervention, comparison, outcome (PICO) question

The PICO question set was as follows: “What is the effect of micrografting technology in the regeneration of epithelial or nonepithelial tissue loss compared with nontreatment or controlled intervention in adults?”

### Inclusion and exclusion criteria

Study designs eligible for inclusion were randomized clinical trials, controlled clinical trials, pilot trials, and observational studies (including cohort, case–control, cross-sectional, and case series studies) involving human participants (including prospective and retrospective studies). Case reports, in vitro studies, animal studies, reviews, and letters to the editor were excluded.

### Information sources and search strategy

A systematic electronic search was conducted until 15 July 2024 using seven electronic databases, namely, Cochrane Central Register of Controlled Trials (CENTRAL), Medline Ovid, Embase Ovid, Cumulative Index to Nursing and Allied Health Literature (CINAHL) EBSCOhost, Web of Science Core Collection, System for Information on Grey Literature in Europe (SIGLE), and Bielefeld Academic Search Engine (BASE). The search strategy is illustrated in Supplementary Table 1. No language or year limits were imposed.

### Selection process

Two reviewers (RA and SK) independently screened the titles and abstracts of the studies for inclusion based on predefined eligibility criteria. Two reviewers (RA and YY) independently assessed the full text of the studies for inclusion. Disagreement was resolved via discussion with a third reviewer (FD).

### Data extraction and risk of bias assessment

**Figure 2.**
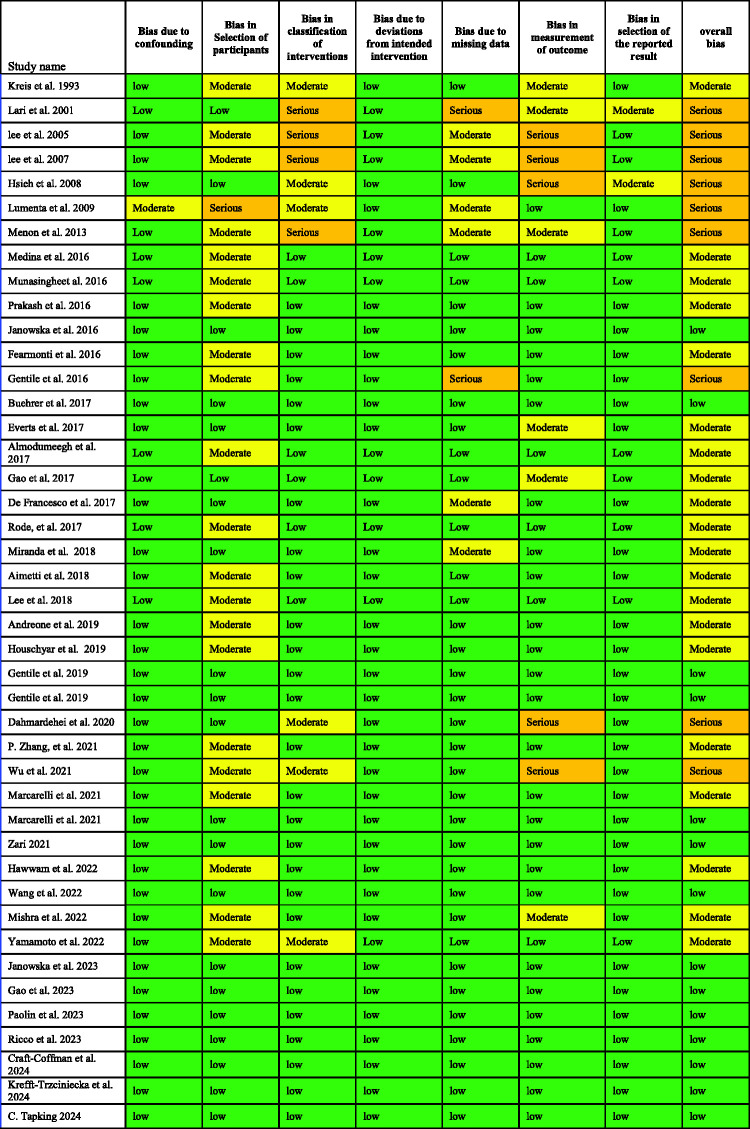

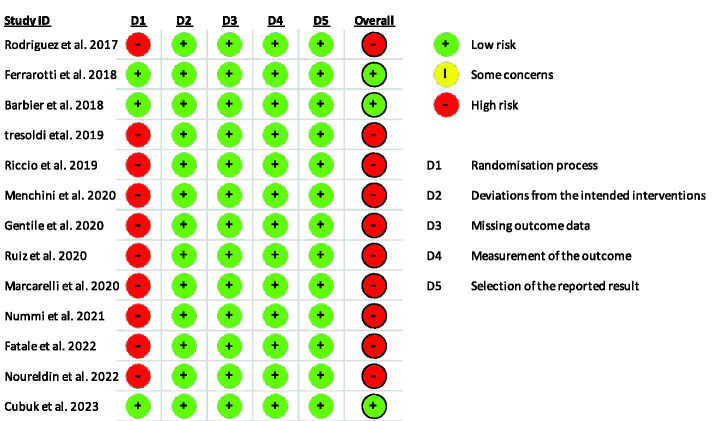
Risk of bias (RoB) assessment for (a) nonrandomized studies and (b) randomized studies.

One reviewer (RA) performed data extraction, which was subsequently checked by a second reviewer (YY). Data were entered into an Excel spreadsheet and included all relevant outcome measures, such as micrografting type, the condition being treated, participants’ characteristics, outcome, and healing time. The studies were grouped according to study design and number of interventions. The risk of bias in non-randomized studies of interventions (ROBINS-I) tool was used for nonrandomized studies (including observational studies for interventions) and risk of bias (RoB) 2 tool for randomized trials^9,10^ (Figure 2).

### Synthesis methods

All available evidence was analyzed using descriptive and quantitative methods. The pooled mean difference and 95% confidence intervals (CIs) for various factors, such as graft survival, hospital length of stay (in days), wound healing time, and surgery time (in hours), were calculated using Stata 17 with random-effect models. To assess the robustness of our findings, sensitivity analyses were performed by excluding one study with a small sample size in the analyses. Results followed the same pattern but with reduced sample size and statistical significance. Furthermore, a new graph was created, as shown in Supplementary Figure 1.

## Results

### Study characteristics

A total of 1785 studies were identified through electronic search after duplicate removal.^8^ Following the screening of titles and abstracts, 245 studies were deemed eligible for full-text assessment. Of these, 55 studies consisting of 2 case–control studies, 13 randomized controlled trials, and 40 nonrandomized studies were included in a qualitative analysis. Most studies were deemed to have moderate-to-severe bias after quality assessment.

The studies were categorized based on the disease, condition, or type of tissue loss for which micrografting technology was applied (Tables 1–4). Treatment for burns was the most common indication for the use of micrografts (24 studies). Four studies were suitable for inclusion in meta-analysis: three studies reporting graft survival, two studies reporting the hospital length of stay (in days), four studies reporting wound healing, and four studies reporting total time of surgery. Furthermore, micrografting technology was used for treating ulcers and wounds in 10 studies, vitiligo in 3 studies, androgenetic alopecia in 7 studies, and cartilage and bone defects in 3 studies as well as for performing coronary artery bypass graft surgery (CABG) in 1 study. In dentistry, micrografting technology was used for sinus lift (three studies), socket preservation technique (two studies), and intrabody defects (two studies).

**Table 1.** Observational studies with one intervention and no control.

Author, year	Study type	Intervention	Age (years)/sex/number/graft number	Outcome		Healing time
Burn						
Kreis et al. 1993^7^	Case series	Modified Meek technique	4–52/3F, 7M/10/16	% TBSA	64 ± 14.9	5 weeks
				% Full skin thickness	47 ± 16.5	
				% TBSA grafted	10 ± 3.2	
				Hospital length of stay (days)	97.9 ± 23.4	
				Total time of the surgery	NR	
				% Graft uptake	92 ± 18.8	
Lari et al. 2001^11^	Case series	Modified Meek technique	13–42/4F, 3M/7/17	% TBSA	74 ± 12.4	5 weeks
				% Full skin thickness	56% (33%–78%)	
				% TBSA grafted	16% (15%–20%)	
				Hospital length of stay (days)	NR	
				Total time of the surgery	NR	
				% Graft uptake	90	
Hsieh et al. 2008^12^	Case series	Modified Meek technique	8–80/26M, 11F/37/68	TBSA	72.9% (40%–97%)	NR
				% Full skin thickness	10%–90% (41%)	
				% TBSA grafted	13.8 (8–25)	
				Hospital length of stay (days)	NR	
				Total time of the surgery	NR	
				Graft uptake	90–95	
Menon et al. 2013^14^	Retrospective chart review	Combined modifiedMeek technique and CEA	4–12/NR/7/NR	TBSA	45.7 ± 15.6	NR
				% Full skin thickness	NR	
				% TBSA grafted	NR	
				Hospital length of stay (days)	51 ± 11	
				Total time of the surgery	NR	
				% Graft uptake	NR	
Medina et al. 2016^15^	Retrospective study	Modified Meek technique	35.4 ± 5.2/9M, 1F/10/NR	TBSA	68 ± 9.2	NR
				% Full skin thickness	NR	
				% TBSA grafted	43.4% ± 11.6%	
				Hospital length of stay (days)	86 ± 30	
				Total time of the surgery	6.57 ± 1 h	
				% Graft uptake	74.4 (37.5–100)	
Munasinghe et al. 2016^26^	Retrospective chart review	Modified Meek technique	23–64/7M, 4F/11/NR	TBSA	56.75 ± 19.6	NR
				% Full skin thickness	NR	
				% TBSA grafted	16 ± 9.9	
				Hospital length of stay (days)	98 (44–167)	
				Total time of the surgery	NR	
				% Graft uptake	87 ± 27.7	
Almodumeegh et al. 2017^16^	Retrospective chart review	Modified Meek technique	18–92/34M, 33F/67/148	TBSA	65% (50%–87%)	NR
				% Full skin thickness	52% (40%–81%)	
				% TBSA grafted	20% (15%–25%)	
				Hospital length of stay (days)	27 (4–62) days	
				Total time of the surgery	NR	
				% Graft uptake	60–90	
Rode et al. 2017^18^	Cohort study	Modified Meek technique	3 months–11 year/NR/35/NR	TBSA	49.6% (15%–86%)	1 month
				% Full skin thickness	NR	
				% TBSA grafted	29.34% (5%–82%)	
				Hospital length of stay (days)	NR	
				Total time of the surgery	NR	
				% Graft uptake	88.1 (60–100)	
Lee et al. 2018^19^	Retrospective review	Modified Meek technique	2–11/2F, 10M/12/34	TBSA	35.4% ± 19%	NR
				% Full skin thickness	26.2% (10%–65%)	
				% TBSA grafted	NR	
				Hospital length of stay (days)	58 ± 34.5	
				Total time of the surgery	NR	
				% Graft uptake	87.1 ± 14.5	
Houschyar et al. 2019^20^	Retrospective analysis	Modified Meek technique	15–66/3F, 9M/12/NR	TBSA	54.3% (31%–77%)	NR
				% Full skin thickness	NR	
				% TBSA grafted	NR	
				Hospital length of stay (days)	54 (28–70)	
				Total time of the surgery	NR	
				% Graft uptake	83	
P. Zhang et al. 2021^22^	Retrospective study	Modified Meek technique	38.9 ± 14.0/68M, 15F/83/NR	TBSA	64.0 ± 18.1	NR
				% Full skin thickness	34.5 ± 19.5	
				% TBSA grafted	24.3% ± 8.3%	
				Hospital length of stay (days)	NR	
				Total time of the surgery	NR	
				% Graft uptake	NR	
Lee et al. 2005^30^	Case series	Fly-paper technique postage stamp skin autografting	20–82/3F, 3M/6	TBSA	52.6 ± 26.6	29.3 ± 4.4 days
				% Full skin thickness	NR	
				% TBSA grafted	23.8 ± 11.3	
				Hospital length of stay (days)	NR	
				Total time of the surgery	NR	
				% Graft uptake	NR	
Lee et al. 2007^31^	Case series	Fly-paper technique postage stamp skin autografting	27–74/3M, 2F/5/NR	TBSA	30 ± 15	26 ± 1.8 days
				% Full skin thickness	NR	
				% TBSA grafted	18.4 ± 6.62	
				Hospital length of stay (days)	NR	
				Total time of the surgery	NR	
				% Graft uptake	90	
Andreone et al. 2019^33^	Retrospective review	Rigenera + platelet-rich fibrin	22–46/5M/5/NR	TBSA	21.3 ± 14.7	NR
				% Full skin thickness	NR	
				% TBSA grafted	NR	
				Hospital length of stay (days)	**52.4 ± 41.8**	
				Total time of the surgery	NR	
				% Graft uptake	97.4 ± 0.9	
Gao et al. 2023^27^	Retrospective review	Modified Meek technique	26–53/6M, 1F/7/NR	TBSA	89 ± 4.8	84–162 days
				% Full skin thickness	NR	
				% TBSA grafted	NR	
				Hospital length of stay (days)	127 ± 53	
				Total time of the surgery	NR	
				% Graft uptake	81	
Craft-Coffman et al. 2024^28^	Retrospective review	Combined modified Meek technique and CEA	65.6 ± 9.44/12M, 3F/15/NR	TBSA	65.6 ± 9.44	NR
				% Full skin thickness	NR	
				% TBSA grafted	NR	
				Hospital length of stay (days)	95.2 ± 35.4	
				Total time of the surgery	NR	
				% Graft uptake	82 ± 9.4	
Tapking et al. 2024^29^	Retrospective review	Modified Meek technique	45.7 ± 19.9/63M, 10F/73	TBSA	60.0 ± 17.8	NR
				% Full skin thickness	NR	
				% TBSA grafted	42.9 ± 13.4%	
				Hospital length of stay (days)	82.5 ± 57.9	
				Total time of the surgery	NR	
				% Graft uptake	75.8 ± 14.7	
Ulcer and wound						
Prakash et al. 2016^34^	Case series	CelluTome epidermal harvesting system	32–70/9F, 9M/18/NR	Wound duration (months)	36.8 ± 48.5	3.7 ± 1.8 weeks
				Wound size	<49 cm^2^	
				Re-epithelialization %	88.90	
R. M. Fearmonti et al. 2016^35^	Case series	CelluTome epidermal harvesting system	61.6/15M, 7F/22/23	Wound duration (months)	NR	10 ± 7.5 weeks
				Wound size	NR	
				Re-epithelialization %	88.1 ± 16.3	
Everts et al. 2017^37^	Case series	CelluTome epidermal harvesting system	64.1 ± 15.6/58F, 22M/78/NR	Wound duration (months)	13.2 ± 25.2 months (range: 0.3–180 months)	10.0 ± 7.3 weeks
				Wound size	0.15 ± 0.21 cm^2^	
				Re-epithelialization %	84.6	
Janowska et al. 2023^38^	Case series	CelluTome epidermal harvesting system	49–91/8F, 7M/15/NR	Wound size	6.3 ± 8.1 cm^2^	3 weeks
				Wound size after treatment	3.7 ± 7.6 cm^2^	
				Wound duration (weeks)	6	
De Francesco et al. 2017^39^	Case series	Rigenera + collagen sponge	64 ± 5/NR/30/NR	Wound duration (weeks)	from <2 to >12	4 weeks
				Wound size	10 cm^2^	
				Wound size after treatment	0	
				VAS score	5	
				VAS score after treatment	2	
Miranda et al. 2018^40^	Retrospective review	Rigenera + collagen sponge	57–82/11F, 4M/ 15	Wound size	6–120 cm^2^	9.2 ± 4 weeks
				Wound duration	12–48 weeks	
				Wound reduced at 2 weeks	37.33% ± 19.35%	
				Re-epithelialization %	86.70	
Vitiligo						
Janowska et al. 2016^44^	Case series	CelluTome epidermal harvesting system	23–67/3F, 2M/5/NR	VASI	NR	1–3 months
				Repigmentation good, n = 4	50%–75%	
Androgenetic alopecia						
Zari 2021^51^	Retrospective cohort study	Rigenera	18–65/113F, 27M/140	Hair density (N/cm^2^)	162.1 ± 37.1	2 months
				Hair density after treatment	167.8 ± 37	
Hawwam et al. 2022^52^	Case series	Rigenera	30–45/F/20	Hair density/cm^2^	184.1 ± 40.9	3 months
				Hair density after treatment	190.4 ± 38.8	
Cartilage and bone defects						
Gentile et al. 2016^54^	Case series	Rigenera + PRP	23–67/NR/11/NR			6 months
Marcarelli et al. 2021^55^	Case series	Rigenera	43–69/4F, 4M/8	VAS score	5.5 ± 1.6	6 months
				VAS score after treatment	1.8 ± 0.7	
				OKS	28.4 ± 6	
				OKS after treatment	40.8 ± 6.2	
Intrabony defects						
Aimetti et al. 2018^64^	Case series	Rigenera	51.2 ± 6.1/5F, 6M/11/NR	PD	8.2 ± 1.3	12 months
				PD after treatment	3.2 ± 0.9	
				CAL	10.7 ± 1.9	
				CAL after treatment	6.0 ± 1.2	
Sinus left						
Paolin et al. 2023^60^	Case series	Rigenera	36–71/3F, 2 M/5/NR	MBL baseline	0.35 ± 0.49	6 months
				MBL at 6 months	0.55 ± 0.52	

TBSA: total body surface area; VAS: Visual Analog Scale; OKS: Oxford Knee Score; VASI: Vitiligo Area Scoring Index; PD: pocket depth; CAL: clinical attachment loss; MBL: marginal bone level; CEA: cultured epithelial autograft; PRP: platelet-rich plasma; NR: not recorded.

**Table 2.** Clinical trials with one intervention and no control.

Author, year	Study type	Intervention	Age (years)/sex/number/graft number	Outcome		Healing time
Vitiligo						
Menchini et al. 2020^46^	Clinical study	Rigenera + NBUVB phototherapy	37.6 ± 12.8/17M, 3F/20/NR	Percentage of vitiligo area, n = 20	59.10%	6 months
				Percentage of vitiligo area after 6 months, n = 20	27.70%	
				Repigmentation very good, n = 6	(≥75%)	
				Repigmentation good, n = 4	51%–75%	
				Repigmentation fair, n = 10	26%–50%	
Androgenetic alopecia						
Ruiz et al. 2020^50^	Clinical study	Rigenera	NR/NR/100/NR	Hair density (N/cm^2^) before treatment	15.2	2 months
				Hair density (N/cm^2^) after treatment	48.5	
				Mean hair density after	30.0% ± 3.0%	
Krefft-Trzciniecka et al. 2024^53^	Clinical study	Rigeneran	40 ± 12/23F/23/115	VAS before treatment	5.2 ± 2.2	6 months
				VAS after treatment	7.2 ± 2.1	
Wound and ulcers						
Riccio et al. 2019^42^	Clinical study	Rigenera	34–74/38F, 32M/70	Wound duration (weeks)	Range: 2–18	5–12 weeks
				Surface area at day 0	14 (range: 7–28) cm^2^	
				Surface area at day 7	11.6 ± 2.3 cm^2^	
				Surface area at day 21	4.5 ± 2.1 cm^2^	
				Surface area at day 48	**0 ± 1.3 cm^2^)**	
				VAS score before treatment	6 (9–4)	
				VAS at 2 months of follow-up	3.4 (5–2)	
Riccio et al. 2023^43^	Clinical study	Hy-Tissue Micrograft Technology	25–70/5F, 6M/11	Surface area at day 0	10–45	2 months
				Wound Bed Scale (WBS) score at baseline	5.27	
				WBS score at 2 months	11	
Cartilage and bone defects						
Marcarelli et al. 2020^56^	Clinical study	Rigenera	40–63/20M/20/NR	Harris hip score	68	2 months
				Harris hip score after treatment	84	
				Oxford Hip Score	28.1 ± 6.5	
				Oxford Hip Score after treatment	37.4 ± 9.5	

WBS: Wound Bed Scale; VAS: Visual Analog Scale; NR: not recorded.

**Table 3.** Observational studies with two or more interventions.

Author, year	Study type	Intervention	Age (years)/sex/number/graft number	Groups	Outcome		Healing time
Burn							
Lumenta et al. 2009^13^	Cohort study	Modified Meek technique	26–65/9M, 1F/10/196	Meek, n = 6	TBSA	71.6% ± 11.0%	4–5 weeks
					% Full skin thickness	59.5 ± 17.9	
					% TBSA grafted	NR	
					Hospital length of stay (days)	85.7 ± 14.8	
					Total time of the surgery	NR	
					% Graft uptake	85	
				Non-Meek, n = 4	TBSA	67.0 ± 12.0	
					% Full skin thickness	NR	
					% TBSA grafted	NR	
					Hospital length of stay (days)	84.3 ± 26.1	
					Total time of the surgery	NR	
					% Graft uptake	NR	
Wu et al. 2021^23^	Retrospective review	Modified Meek technique	12–31/12M, 12F/24/NR	Meek, n = 14	TBSA	71.5 (50–92)	NR
					% Full skin thickness	47.2 (25–67.5)	
					% TBSA grafted	11.3 (4–32)	
					Hospital length of stay (days)	160.4 (72–301)	
					Total time of the surgery	NR	
					% Graft uptake	72.9	
				Non-Meek, n = 10	TBSA	53.8 (42–75)	
					% Full skin thickness	13.6 (0–40)	
					% TBSA grafted	NR	
					Hospital length of stay (days)	74.8 (27–123)	
					Total time of the surgery	NR	
					% Graft uptake	NR	
Mishra et al. 2022^24^	Retrospective review	Modified Meek technique	40–97/6F, 5M/11/NR	Meek, n = 6	TBSA	14 ± 2.6	54.8 ± 22.3
					% Full skin thickness	NR	
					% TBSA grafted	12.5 ± 2	
					Hospital length of stay (days)	24.8 ± 9.2	
					Total time of the surgery	2.02 ± 0.5	
					% Graft uptake	82.3 ± 13.7	
				Mesh, n = 5	TBSA	13.2 ± 5.5	62 ± 15.3
					% Full skin thickness	NR	
					% TBSA grafted	8.5 ± 4.5	
					Hospital length of stay (days)	53.6 ± 35.1	
					Total time of the surgery	2.3 ± 1	
					% Graft uptake	79 ± 22.4	
Yamamoto et al. 2022^32^	Retrospective case series	Rigenera	20–82/3F, 3M/6/NR	Rigenera + meshed split-thickness skin grafts	TBSA	8.5 ± 3.5	34.5 ± 6.7 (range: 28–46) days after injury and 18.2 ± 7.6 (range: 7–28) days
					% Full skin thickness	NR	
					% TBSA grafted	NR	
					Hospital length of stay (days)	NR	
					Total time of the surgery	NR	
					% Graft uptake	NR	
				Rigenera	TBSA	8.5 ± 3.5	
					% Full skin thickness	NR	
					% TBSA grafted	NR	
					Hospital length of stay (days)	NR	
					Total time of the surgery	NR	
					% Graft uptake	NR	
Ulcer and wound							
Buehrer et al. 2017^36^	Prospective controlled study	CelluTome epidermal harvesting system	34–88/12F, 8M/20/NR	Epidermal micrografts recipient site	Hemoglobin concentration	90⋅4 ± 10⋅2	4 weeks
					Oxygen saturation	69⋅0 ± 24⋅0	
					(NRS)	0⋅8 ± 1⋅2	
				Mesh graft donor site	Hemoglobin concentration	94⋅0 ± 12⋅3	
					Oxygen saturation	71⋅2 ± 21⋅8	
					(NRS)	1⋅1 ± 0⋅75	
Vitiligo							
Wang et al. 2022^45^	Retrospective review	CelluTome epidermal harvesting system	31.38 ± 13.56/72F, 46M/118/NR	ABEM, n = 56	VASI	96.25 ± 8.59	
					VASI after treatment	48.30 ± 28.16	
					Repigmentation very good	(≥75%)	
					Repigmentation good	51%–75%	
					Repigmentation fair	(26%–50%)	
				SBEG, n = 62	VASI	96.69 ± 9.71	
					VASI after treatment	68.6 ± 26.16	
					Repigmentation very good	47 (76%)	
					Repigmentation good	5 (8%)	
					Repigmentation fair	1 (2%)	
Androgenetic alopecia							
Gentile 2019^47^	Retrospective observational case series	Mechanical fragmentation and centrifugation of scalp biopsy samples	21–70/12F, 23M/35/NR	HD-AFSCs	Mean hair density	33% ± 7.5%	6 months
				Placebo (saline solution).	Mean hair density	<1%	
Gentile et al. 2019^49^	Rretrospective observational case series	Rigeneran	NR	HF-MSCs, n = 21	Mean hair density	30% ± 5.0%	3 months
				A-PRP, n = 57	Mean hair density	31% ± 2.0%	
				Placebo	Mean hair density	1%	

TBSA: total body surface area; NRS: numeric rating scale; VASI: Vitiligo Area Scoring Index; HF-MSCs: hair follicle-derived mesenchymal stem cells; A-PRP: autologous platelet-rich plasma; NR: not recorded; ABEM: automated blister epidermal micrograft; SBEG: suction blister epidermal graft; HD-AFSCs: human intra- and extra-dermal adipose tissue-derived hair follicle stem cells.

**Table 4.** Controlled clinical trial and case–control studies.

Author, year	Study type	Intervention	Age (years)/sex/number/graft number	Groups	Outcome		Healing time
Burn							
Gao et al. 2017^17^	Clinical study	Modified Meek technique	30–50/75M, 30F/104/NR	Meek, n = 35	%TBSA	73.72 ± 10.48	30.78 ± 3.18 days
					% Full skin thickness	NR	
					% TBSA grafted	NR	
					Hospital length of stay (days)	NR	
					Total time of the surgery	3.14 ± 0.64	
					% Graft uptake	91.76% ± 1.5%	
				Stamp, n = 34	%TBSA	71.27 ± 10.06	46.26 ± 9.93 days
					% Full skin thickness	NR	
					% TBSA grafted	NR	
					Hospital length of stay (days)	NR	
					Total time of the surgery	3.26 ± 0.66	
					% Graft uptake	76.24% ± 3.97%	
				Microskin, n = 35	%TBSA	73.51 ± 10.29	48.49 ± 7.53 days
					% Full skin thickness	NR	
					% TBSA grafted	NR	
					Hospital length of stay (days)	NR	
					Total time of the surgery	3.18 ± 0.68	
					% Graft uptake	73.55% ± 2.85%	
Dahmardehei et al. 2020^21^	Case–control study	Modified Meek technique	19–54/18M, 2F/20/40	Meek, n = 20	%TBSA	36.9% ± 16.6%	2.8 ± 2.5 months
					% Full skin thickness	NR	
					% TBSA grafted	39%	
					Hospital length of stay (days)	84 ± 75	
					Total time of the surgery	0.44 ± 0.09	
					% Graft uptake	85%	
				Mesh, n = 20	%TBSA	NR	5.0 ± 2.1 months
					% Full skin thickness	NR	
					% TBSA grafted	30%	
					Hospital length of stay (days)	150 ± 63	
					Total time of the surgery	0.53 ± 0.12	
					% Graft uptake	75%	
Noureldin et al. 2022^25^	Randomized case–control	Modified Meek technique	1–16/27M, 13F/40	Meek, n = 20	%TBSA	19.85 ± 7.68	27.11 ± 12.23 days
					% Full skin thickness	13.00 ± 3.51	
					% TBSA grafted	NR	
					Hospital length of stay (days)	27.11 ± 12.23	
					Total time of the surgery	3 ± 1.14	
					% Graft uptake	84.25 ± 8.93	
				Mesh, n = 20	%TBSA	16.6 ± 8	33.50 ± 18.79 days
					% Full skin thickness	11.55 ± 3.55	
					% TBSA grafted	NR	
					Hospital length of stay (days)	33.50 ± 18.79	
					Total time of the surgery	1.3 ± 0.14	
					% Graft uptake	71.5 ± 17.3	
Ulcer and wound							
Tresoldi et al. 2019^41^	Prospective RCT	Rigenera + Integra dermal regeneration template	53–93/4F, 16M//20/24	Integra + Rigenera, n = 11	Wound size	7.06–12.54 cm^2^	4 weeks
					Re-epithelialization rate at 4 weeks	15.14% (12.42%–22.03%)	
				Integra, n = 12	Wound size	7.06–12.54 cm^2^	
					Re-epithelialization rate at 4 weeks	12.98% (10.40%–17.61%)	
Androgenetic alopecia							
Gentile et al. 2020^48^	Placebo controlled, randomized study	Mechanical fragmentation and centrifugation of scalp biopsy samples	NR/17M, 10F/27/NR	HF-MSCs	Mean hair density	23.3 hairs increase	14 months
				Placebo (saline solution)	Mean hair density	0.7 hairs per cm^2^ decrease	
Ischemic heart disease							
Nummi et al. 2021^57^	Nonrandomized open-label study	Rigenera	63–76/10M, 2F/NR	CABG + AAMs, n = 6	ECG: Q Wave	1 (17%)	3 months
					ECG: Q Wave after treatment	2 (33%)	
					Left atrium (mm)	44 (40–56)	
					Left atrium (mm) after treatment	44.5 (38–50)	
				CABG, n = 4	ECG: Q Wave	3 (50%)	
					ECG: Q Wave after treatment	4 (67%)	
					Left atrium (mm)	45 (32–50)	
					Left atrium (mm) after treatment	48 (30–49)	
Sinus lift							
Rodriguez et al. 2017^58^	Clinical study	Rigenera	45–64/12F, 12M/24/NR	Alos + micrografts (A)	Vital mineralized tissue	58.5 ± 2.5	4 months
					Non-mineralized tissue	41.4 ± 5.6	
				Alos (B)	Vital mineralized tissue	20.2 ± 3.1	
					Non-mineralized tissue	5.5 ± 1.6	
				Bio-Oss ©	Vital mineralized tissue	48 ± 2.5	
					Non-mineralized tissue	20.5 ± 3.1	
Fatale et al. 2022^59^	Clinical study	Rigenera	30–80/80/7f 17 M/24/NR	Beta-tricalcium phosphate (80%), hydroxyapatite (20%), tetracycline, collagen particles, and MSCs	Type 1 mature bone	44.45%	3 months
					Type 2 osteoid tissue	7.04%	
				Beta-tricalcium phosphate (80%), hydroxyapatite (20%), tetracycline, and collagen particles	Type 1 mature bone	27.24%	
					Type 2 osteoid tissue	10.86%	
Socket preservation technique							
Barbier et al. 2018^61^	RCT	Rigenera	18–30/22F, 8M/30/60	ADPMSC + collagen, n = 30	Bone density at 6 months	507.8 ± 353.8	6 months
				collagen only, n = 30	Bone density at 6 months	583.66 ± 389.5	
Cubuk et al. 2023^62^	RCT	L-PRF membranes +Rigenera	22–60/4F, 2M/6/NR	L-PRF + DPSC	PPD (mm) at baseline	4.65 ± 0.69	6 months
					PPD (mm) at 6 months	3.11 ± 0.58	
					CAL (mm) at baseline	2.77 ± 0.75	
					CAL (mm) at 6 months	0.65 ± 0.32	
				L-PRF	PPD (mm) at baseline	4.27 ± 0.67	
					PPD (mm) at 6 months	3.08 ± 0.94	
					CAL (mm) at baseline	2.85 ± 1.14	
					CAL (mm) at 6 months	0.62 ± 1.04	
Intrabony defects							
Ferrarotti et al. 2018^63^	RCT	Rigenera	39–69/14F, 13M/29/NR	Rigenera + collagen sponge	PD	8.3 ± 1.2	12 months
					PD after treatment	3.4 ± 0.9	
					IBD	6.4 ± 1.4	
					IBD after treatment	2.5 ± 0.7	
					CAL	10.0 ± 1.6	
					CAL after treatment	5.5 ± 1.1	
				Collagen sponge	PD	7.9 ± 1.3	
					PD after treatment	4.5 ± 1.0	
					IBD	5.6 ± 1.0	
					IBD after treatment	4.0 ± 0.8	
					CAL	9.4 ± 1.5	
					CAL after treatment	6.5 ± 1.2	

TBSA: total body surface area; HF-MSCs; hair follicle-derived mesenchymal stem cells; CABG: coronary artery bypass graft surgery; AAMs: atrial appendage micrografts; ECG: electrocardiogram; ADPMSCs: autologous dental pulp mesenchymal stem cells; L-PRF: leukocyte- and platelet-rich fibrin; PPD: periodontal pocket depth; IBD: intrabony defect depth; RCT: randomized controlled trial; NR: not recorded.

## Outcome results by condition: burns

### The Meek technique

In 20 studies,^7,11–29^ the Meek technique was used to treat a total of 485 patients. The total body surface area (TBSA) percentages ranged from 14% to 92%, with the percentage of full skin thickness injuries ranging from 2.5% to 78%. The TBSA grafted per procedure using the Meek technique varied from 5% to 44%. Heterogeneity and inconsistent reporting of study outcomes were common among all studies, which were mostly deemed to have moderate-to-severe risk of bias.

#### Graft uptake

**Figure 3.**
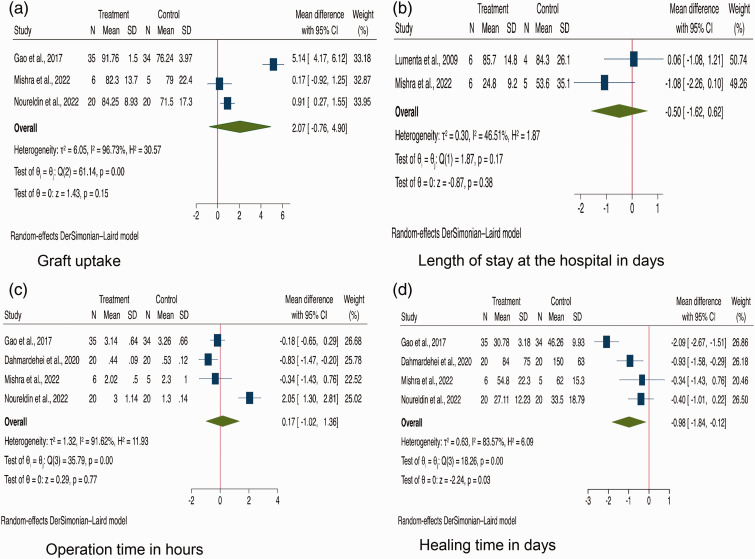
Forest plot of the Meek technique compared with control. (a) Graft uptake, (b) Length of stay at the hospital, (c) Operation time, and (d) healing time.

In 17 studies, the percentages of graft uptake were reported, which ranged from 73% to 92%.^7,11–13,15–21,24–29^ Quantitative analyses of the three available studies revealed no statistically significant difference between the Meek and non-Meek techniques in terms of graft uptake (weighted mean difference (WMD): 2.07, 95% CI: −0.76 to 4.90, p* *=* *0.15), with a high level of heterogeneity (I^2^ = 96) and moderate-to-severe risk of bias (Figure 3(a)).

#### Length of stay at the hospital in days

Thirteen studies reported an average hospital stay ranging from 25 to 160 days.^7,13–16,19,20,23,24,26–29^ Two studies included in the meta-analysis showed no statistically significant difference between the Meek and non-Meek techniques with regards to length of stay at the hospital in days (WMD: −0.50, 95% CI: −1.62 to 0.62, p* *=* *0.38), with a medium level of heterogeneity (I^2^ = 46.51%) and overall moderate risk of bias (Figure 3(b)).

#### Operation time in minutes

The total operative time was reported in five studies, ranging from 44 minutes to 7 hours.^15,17,21,24,25^ In the meta-analysis of four studies, there was no statistically significant difference between the Meek and non-Meek techniques (WMD: 0.17, 95% CI: −1.02 to 1.36, p* *=* *0.77), with a high level of heterogeneity (I^2^ = 91.62%) and moderate risk of bias (Figure 3(c)).

#### Healing time

Ten studies reported the average healing time of skin grafts, which ranged from 7 days to 3 months.^7,11,13,15,17,18,21,23–25^ A meta-analysis of four studies revealed that the Meek technique resulted in shorter healing times than the non-Meek technique (WMD: −0.98, 95% CI: −1.84 to −0.12, p* *=* *0.03), although there was a high level of heterogeneity (I^2^ = 83.57%) and moderate risk of bias (Figure 3(d)).

### Fly-paper technique postage stamp skin autografting

Two studies utilized the fly-paper technique, which is an adaptation of the Meek technique but with different tools that produce larger micrografts measuring 5 × 5 mm, and compared it with Meek’s 3 × 3 mm micrograft size for skin grafting.^30,31^ No quantitative analyses were performed, and a moderate risk of bias was observed.

#### Healing time

A total of 11 patients underwent this procedure, and their healing time varied from 26 to 29 days. No quantitative analyses were performed, and a moderate risk of bias was observed.

### Rigenera

In two studies, the effectiveness of Rigenera technology in treating burns was analyzed.^32,33^ One study compared the use of Rigenera + meshed split-thickness skin graft with Rigenera alone. This study revealed that the combined treatment resulted in a shorter healing time.^32^ In another study, a treatment using Rigenera combined with platelet-rich fibrin (PRF) resulted in a graft uptake of 97%; however, no comparison was performed.^33^ No quantitative analyses were performed, and a moderate risk of bias was observed.

## Outcome results by condition: wounds and ulcers

### CelluTome epidermal harvesting system

In total, 153 patients aged 32–91 years were included in 5 studies.^34–38^ The wounds ranged in duration from 6 weeks to 15 years and in size from 0.15 to 49 cm^2^. Healing time varied from 3 to 10 weeks. No quantitative analyses were performed, and a moderate risk of bias was observed.

### Rigenera

In four studies, 135 patients were treated with Rigenera technology for wounds ranging in duration from less than 2 weeks to 48 weeks and in size from 6 to 120 cm^2^. The wounds were successfully healed within 5–12 weeks.^39–42^ No quantitative analyses were performed, and a moderate risk of bias was observed.

### Hy-Tissue micrograft technology

In a study involving 11 patients who had nonresolving wounds (at least 3 months after conventional therapy), the patients were treated with the Hy-Tissue micrografting technique.^43^ All patients in the study experienced better wound healing within 6 months of micrografting.

## Outcome results by condition: vitiligo

### CelluTome epidermal harvesting system

In 2 studies involving 39 patients, the CelluTome technique was utilized to treat vitiligo. The repigmentation process took 1–6 months for completion. Of the 39 patients included, 23 had a good degree of repigmentation (51%–75%), while 12 patients had a fair degree of repigmentation (26%–50%).^44,45^ No quantitative analyses were performed, and a moderate risk of bias was observed.

### Rigenera

Only 1 study involving 20 patients was retrieved; these patients received a combination of phototherapy and Rigenera treatment. Four patients achieved a good degree of repigmentation (51%–75%), while 10 patients had a fair degree of repigmentation (26%–50%).^46^ No quantitative analyses were performed, and a moderate risk of bias was observed.

## Outcome results by condition: androgenetic alopecia

### Mechanical fragmentation and centrifugation of scalp biopsy samples

In 2 studies, 62 patients underwent micrografting with stem cells from human hair follicles (HFs) and adipose tissues; these patients were compared with individuals receiving a placebo. Although the reported time points and hair density measurements varied, there was a noticeable increase in hair density in the test group.^47,48^ No quantitative analyses were performed, and a moderate risk of bias was observed.

### Rigenera

A total of 5 studies, which included 283 patients, showed that using Rigenera improved hair density by approximately 30% or 6 hairs per cm^2^ within 2–6 months.^49–53^ No quantitative analyses were performed, and a moderate risk of bias was observed.

## Outcome results by condition: cartilage and bone defects

### Rigenera

In total, 3 studies involving 39 patients with cartilage and bone defects were conducted. Autologous micrografts were found to stimulate the regeneration of both cartilage and bone with a high degree of variation.^54–56^ No quantitative analyses were performed, and the risk of bias was moderate.

## Outcome results by condition: ischemic heart disease

### Rigenera

One study utilized Rigenera technology and recruited 12 patients who underwent CABG surgery with micrografts (n = 6), compared with CABG surgery alone (n = 6). Although there was no statistically significant difference between the groups, one patient in the control group was readmitted to the hospital due to heart failure. During the 6-month study period, no deaths occurred in the micrografts group, but one patient in the control group died of systolic heart failure.^57^ No quantitative analyses were performed, and the risk of bias was moderate.

## Dental applications in sinus lift

### Rigenera

In 3 studies comprising 53 patients, the use of Rigenera technology led to better bone quality compared with the control groups.^58–60^ No quantitative analyses were performed, and the risk of bias was moderate.

## Dental applications in socket preservation technique

### Rigenera

The analysis involved 2 studies comprising 36 patients. The findings showed that the use of this technology did not result in any statistically significant difference between the test and control groups based on different outcomes.^61,62^ No quantitative analyses were performed, and the risk of bias was moderate.

## Dental applications in intrabony defects

### Rigenera

A total of 30 patients from 2 studies were analyzed. The use of micrografts resulted in better probing pocket reduction and clinical attachment level gain compared with not using micrografts.^63,64^ No quantitative analyses were performed, and the risk of bias was moderate.

## Discussion

This systematic review identified various techniques, including the modified Meek technique, fly-paper technique, postage stamp skin autografting, Rigenera, and CelluTome epidermal harvesting system, and Hy-Tissue. Limited evidence suggests that micrografts lead to faster healing than other grafting techniques. Although micrografting offers promising outcomes in various fields, its application should be considered on a case-by-case basis, considering specific patient needs, clinical conditions, and available resources.

### Burns

This review covers 20 studies reporting on the clinical benefit of using the Meek micrograft technique for treating burns. The percentage of graft uptake after this technique ranged from 73% to 92%, with no substantial difference compared with other techniques. A previous systematic review reported similarly weighted averages (82% ± 7%) of graft uptake across 15 studies.^65^ This review is the only one confirming a potential clinical benefit in reducing the healing time while achieving skin regeneration in the burn area. Among the potential mechanisms underlying this faster wound healing process, it has been proposed that smaller graft sizes can reduce vulnerability to failure and lower the risk of infection from microorganisms, ultimately leading to a quicker healing process and reduced length of stay at the hospital.^15,66^ Another review conducted by Quintero et al. included seven studies and confirmed that patients who underwent the Meek technique had a shorter mean hospital stay compared with those who underwent non-Meek technique (51 vs. 121 days). However, our meta-analysis of two studies did not show a statistically significant difference between the two techniques (WMD: −0.50, 95% CI: −1.62 to 0.62, p* *=* *0.38), with a medium level of heterogeneity (I^2^ = 46.51%).^67^

The Rigenera micrografting technology involves creating a micrograft suspension without using any enzymes or chemicals. A promising aspect of the use of Rigenera micrografting technology is that it is possible to collect mesenchymal stem cells (MSCs), extracellular matrix (ECM), and growth factors without a secondary surgery.^42,59,68^ Although the use of Rigenera in burn treatment is not common, two studies have been conducted to explore its potential effectiveness.^32,33^ In one study, Rigenera was combined with PRF and sprayed onto the burn wound area to increase the yield of grafted cells. This approach was implemented to address the issue of irregular cell distribution and potential loss of grafts when cells are suspended in a low-viscosity solution such as normal saline. Fibrin has also been used to address these problems.^69,70^ In another study, micrografts processed with Rigenera were injected directly into the burn wound area. Both studies demonstrated the beneficial effects of Rigenera.

Micrografting technologies, particularly the Meek technique, have demonstrated effectiveness in treating burns, especially for patients with large burn areas or when donor site availability is limited. This approach reduces healing time and hospital length of stay, with the added benefit of potentially minimizing complications related to donor site morbidity. Based on the existing evidence, it is suggested to use micrografting techniques for partial and full-thickness burns with TBSA involvement of ≥30%, particularly in cases where conventional skin grafting techniques may be challenging due to limited available donor sites.

### Wounds and ulcers

Examination of wound and ulcer healing by analyzing five studies using the CelluTome epidermal harvesting system demonstrated that this system is highly efficient as it has the shortest graft harvest time, fastest donor site healing, and no reported donor site morbidity and can be performed in an outpatient setting without anesthesia.^34–38^ Moreover, being an automated system, it ensures consistent graft quality and is easily reproducible.^71,72^ When assessing Rigenera technology in treating wounds and ulcers,^39–42^ preliminary promising results were observed. Recent research has suggested that using autologous micrografts derived from small pieces of dermal or connective tissue can improve tissue repair for complex wounds after surgery.^73,74^

In vitro studies on Rigenera and Hy-Tissue micrografts have suggested several regenerative mechanisms. Micrografts from Rigenera could enhance the proliferation and migration of fibroblasts and keratinocytes through paracrine signaling, which involves the release of growth factors such as vascular endothelial growth factor, fibroblast growth factor, and transforming growth factor-β1 (TGF-β1). This process improves extracellular matrix remodeling and neovascularization. Research indicates that these micrografts lead to a temporary increase in TGF-β1 expression, facilitating the activation of α-smooth muscle actin-positive myofibroblasts, thereby accelerating collagen deposition and wound contraction. Conversely, Hy-Tissue generates fragmented dermo-epidermal units that exert lasting trophic effects by releasing interleukin (IL)-6, IL-8, insulin-like growth factor-1, and adiponectin, which promote fibroblast and keratinocyte migration and monolayer expansion while reducing the expression of cytotoxicity markers such as LDH. Both technologies utilize autologous tissue-derived growth factor cascades and exosome-mediated signaling to enhance cellular responses critical for wound healing.^43,75,76^

Micrografting technologies such as the Rigenera system and Hy-Tissue showed promise in improving healing times. In particular, in nonhealing wounds or when conventional treatments fail, autologous micrografts could stimulate tissue repair and improve healing outcomes. However, the limited evidence and high heterogeneity of studies in this area suggest that further research is needed to standardize protocols and establish clear clinical guidelines.

### Vitiligo

When assessing the impact of micrografting in the management of vitiligo, only three studies were identified.^44 [Bibr bibr45-03000605251337859]^^–^^46^ One of the studies compared the suction blister epidermal graft and the CelluTome epidermal harvesting system. The results showed that the suction blister epidermal graft had a significantly higher repigmentation rate for stable vitiligo. Only one study combined phototherapy and Rigenera vitiligo^46^ for managing vitiligo, providing limited evidence in defining a potential benefit for patients.

In vitro evidence demonstrated that all epidermal micrografts harvested from the CelluTome system in 12 participants tested positive for Ki-67 at the dermal–epidermal junction. These micrografts preserved collagen type IV, implying that heat and vacuum may partially separate the basement membrane. Notably, all keratinocyte and melanocyte outgrowths were observed on surfaces coated with collagen type I, indicating that these micrografts facilitate cellular expansion. The harvested epidermal micrografts retained their original keratinocyte structure, which is essential for potential re-epithelialization and repigmentation in a wound environment.^77^

The use of micrografting in vitiligo treatment showed potential, although the evidence remained scarce and inconsistent. Micrografting, particularly when combined with phototherapy, could offer benefits in terms of repigmentation, particularly for stable vitiligo. However, due to the limited number of studies, it would be recommended to consider this treatment in cases where traditional therapies have not yielded satisfactory results.

### Androgenetic alopecia

When managing alopecia, two studies^47,48^ documented the progression in the use of micrografts (fragments of the scalp obtained via punch biopsy). This approach did not require cell extension or culturing. Through this procedure, the researchers were able to successfully count cells and identify CD44 + HF-derived MSCs and CD200 + HF-derived epithelial stem cells, as reported in a previous study.^78^ Micrografting should be considered for patients with early-to-moderate stages of androgenetic alopecia who are seeking nonsurgical options to improve hair density, especially when combined with other treatments such as platelet-rich plasma.

Although this review identified some evidence on the use of autologous micrografting to facilitate the regeneration of cartilage and bone tissues, more studies are needed to establish protocols for its use.

### Dental applications

Finally, when evaluating the potential benefit of micrografts in dental applications, only limited evidence with a moderate risk of bias was identified. Although all studies reviewed showed improved clinical outcomes, it was not possible to quantitively assess the magnitude of benefit with regard to alveolar hard tissue regeneration due to the heterogeneity and inconsistency in reporting outcomes. Previous evidence has shown that bone engineering can be achieved with stem cells from dental pulp or periosteum. These stem cells can be acquired through a relatively simple mechanical disaggregation method.^79^ Micrografting technologies such as Rigenera could be used as an adjunct to bone regeneration in dental surgeries, particularly in sinus lift and socket preservation procedures when traditional grafting methods are challenging in terms of graft integration or patient recovery.

### Strengths and limitations

One of the limitations of this review was the high level of heterogeneity of all studies included, which could be attributed to the limited number of studies involved, different tools used for a micrografting procedure, the lack of standardized protocols, and the use of different clinical and biological outcomes that have a direct impact on the generalizability of the evidence obtained in this review. Furthermore, in the meta-analysis concerning heterogeneity within micrografting treatment for burns, high I^2^ values (ranging from 47% to 97%) indicated moderate-to-severe heterogeneity. Key sources of this heterogeneity included the severity of burns and TBSA measures. Varying degrees of burn severity and size may necessitate different treatment protocols, while patient demographics, including age (pediatric vs. adult), comorbidities (e.g. diabetes and malnutrition), and nutritional status, could significantly influence healing and graft integration. Furthermore, burn etiology, such as thermal, chemical, or electrical causes, can result in different wound healing. Variations in micrografting techniques (e.g. expansion ratios), the utilization of adjunct therapies, and methods of donor site harvesting could affect the outcomes. Definitions of success, follow-up duration, and assessment methods (subjective vs. objective) differed among studies, adding to the complexity of the analyses performed. Geographic and institutional variability, including differences in healthcare settings, surgeon experience, and post-grafting wound care, represented another source of heterogeneity in this systematic review. Finally, bias in primary studies could have undermined the reliability of our findings, including confounders’ and selection bias. Furthermore, a robust methodology, prespecified protocol, and overall assessment of a technique irrespective of the clinical outcomes represent the relative strength of this review.

## Recommendation and conclusion

In summary, micrografting is a promising technique in various medical fields, including dental and medical treatments, particularly in reducing healing times and minimizing donor site morbidity. However, the optimal use of these technologies should be based on careful consideration of clinical factors such as wound type, patient health, and available resources. Further high-quality studies are needed to refine protocols, reduce heterogeneity, and establish more robust evidence for the widespread use of micrografting technologies in clinical practice.

## Data Availability

Data can be obtained from the corresponding author upon reasonable request.
